# Immunoassay Development for the Class-Specific Assay for Types I and II Pyrethroid Insecticides in Water Samples

**DOI:** 10.3390/molecules15010164

**Published:** 2010-01-04

**Authors:** Qi Zhang, Wen Zhang, Xiuping Wang, Peiwu Li

**Affiliations:** Oil Crops Research Institute, Chinese Academy of Agricultural Sciences, Wuhan 430062, China; E-Mails: zhangqi521x@yahoo.cn (Q.Z.); zhangwen@oilcrops.cn (W.Z.); xiupinwang@sina.com (X.P.W.)

**Keywords:** hapten, class-specific antibody, sensitivity, immunoassay, pyrethoid insecticides

## Abstract

Five generic haptens of pyrethoid insecticides, which were classified as three types, were designed and synthesized: the first (hapten 1) is for type I pyrethroids without a cyano group, the second (hapten 2 and XQ) for type II pyrethroids with a cyano group, and the third (hapten 4 and 5) for both types of pyrethroids with loss of the ester group. The hapten structures were confirmed by MS and ^1^H-NMR. Hapten 1 and 2 were conjugated with BSA respectively and haptens 1-5 were conjugated with OVA. Four polyclonal antisera were raised against BSA conjugates including a mixture conjugate, and twenty antibody/coating conjugate combinations were selected for studies of assay sensitivity and specificity for pyrethroids. The study revealed the best combination, which showed equal high sensitivities (I_50_ is around 0.02 µg mL^-1^) to both types of pyrethroids. The immunity results suggest that, with a mixture conjugates, a polyclonal antibody against a group of insecticides can be prepared for multi-residue assays.

## Abbreviations

THF: tetrahydrofuran
DMF: dimethyl formamide
NHS: N-hydroxysuccinimide
DCC: N,N-dicyclohexylcarbodiimide
BSA: bovine serum albumin
OVA: ovalbumin
CR: cross-reactivity
ELISA: enzyme-linked immunosorbent assay
TMB: Tetramethylbenzidine
GAM-HRP: peroxidase-labeled goat anti-mouse immunoglobulins
I_50_: concentration giving 50% inhibition of maximum response
LOD: limit of detection
PBS: 150 mM phosphate buffer, pH 7.4
PBST: PBS containing 0.05% (v/v) Tween 20

## Introduction

**Figure 1:**
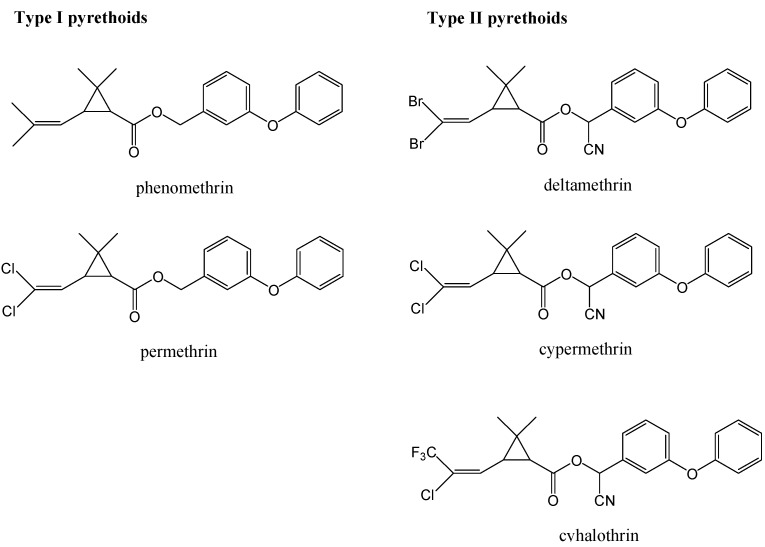
Structures of some synthetic pyrethroids.

Pyrethroid insecticides have been being used widely from agricultural uses [1,2,3,4] to home pest control [5,6] and are effective against a broad range of pests. The synthetic pyrethroids and natural pyrethrins can be divided into two groups of compounds on the basis of their chemical structure and mechanism of action at insect target sites (Figure 1): The type I compounds are simple cyclic alcohol esters of 2,2-dimethyl-3-(2-methyl-1-propenyl)-cyclopropanecarboxylic acid, and the type II compounds are esters of an aryl cyanohydrin [7]. Synthetic pyrethroid residues have been seen in agricultural products [8,9] and food [10]. Lower and lower and lower amounts are being allowed worldwide by regulatory agencies [11]. Many pyrethroids act as a neurotoxin, they are also highly toxic to aquatic life, particularly fish [12,13], so it is very important to develop a rapid, sensitive, specific methods to monitor pyrethroid residues in food products.

Current analytical methods for pyrethroid insecticides involve multistep sample cleanup procedures followed by gas chromatography on instruments equipped with an electron capture detector (GC-EC) [14,15], gas chromatography-mass spectrometry (GC-MS) [16,17], liquid chromatography combined with postcolumn fluorimetry derivatization and fluorescence detection (HPLC-FD) [18,19], or high performance liquid chromatography-mass spectrometry (HPLC-MS) [20]. These methods meet the requirements for sensitivity and accuracy of pyrethroids measurements. On the other hand, they are relatively intensive, time-consuming, expensive, and not particularly suitable for large numbers of samples.

Immunoassays have been considered as a valuable supplement to existing, and rapidly developing, chromatographic techniques, because they have attractive features including high sensitivity and selectivity, rapid detection, and the possibility of analysis of difficult matrices without extensive pre-treatment [21].

When an immunoassay for a single analyte was developed in the past years, people all expected CRs as lower as possible [22,23,24,25,26,27]. With the increase in requests for detection of chemical residues, there has been a gradual focus on total and multi-residues immunoassays. So, with higher CRs, some immunoassays for a type of compounds are also expected and some class specific antibodies against organophosphorus [28,29,30], type I or type II pyrethoid insecticides have been reported [7,31,32]. Here we aimed to develop a combination of coating conjugates and antibodies with equal high sensitivity to both type I and type II pyrethoids.

**Scheme 1:**
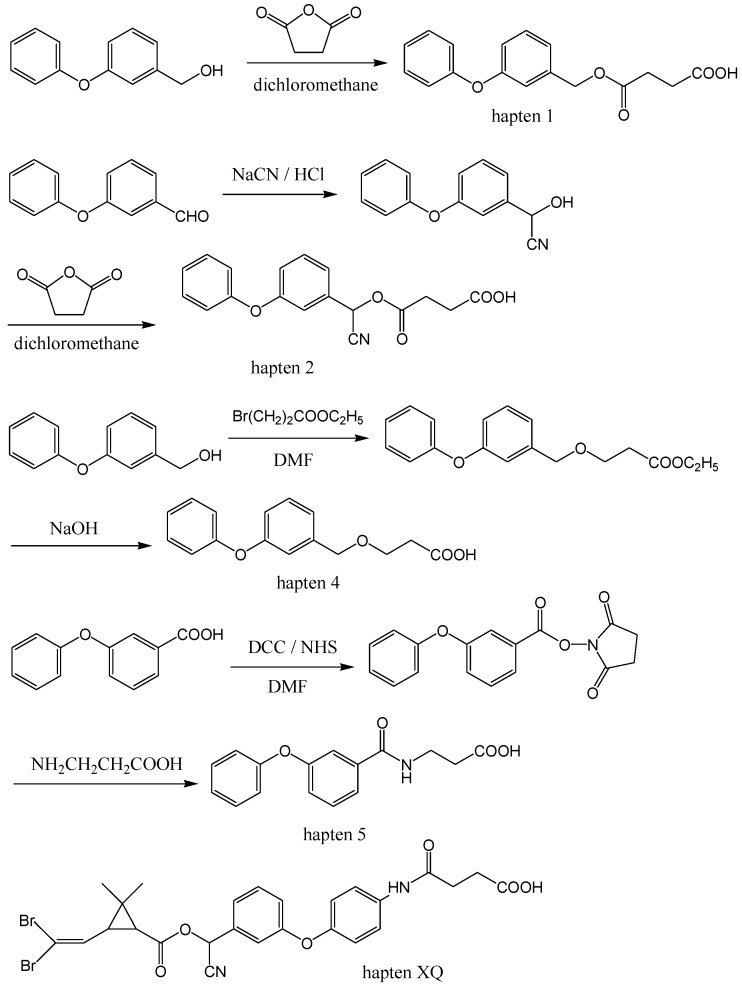
Synthetic schemes for the preparation of generic haptens 1, 2, 4, and 5 and the structure of hapten XQ.

## Results and Discussion

### Hapten design and conjugate verification

Obtaining haptens with proper chemical structures is the key to development of novel antibodies and immunoassays. According to the characteristics of the two types of synthetic pyrethroid pesticides [32], here we designed three types of haptens (Scheme 1): the first (hapten 1) is for type I pyrethroids without a cyano group, the second (hapten 2 and XQ) for type II pyrethroids with a cyano group, and the third (hapten 4 and 5) for both types of pyrethroids with loss of the ester group. Most of these haptens were synthesized simply with just one or two steps and without any rigorous condition.

Through scanning the UV-Vis spectrum, hapten/protein ratios were calculated by measuring the absorbance of the hapten, the protein, and the hapten-protein conjugate at the same wavelength, and the ratios of BSA-hapten 1, BSA-hapten 2, OVA-hapten 1, 2, 4, 5, XQ were 14, 13, 9, 6, 8 and 4. 

Here, hapten 1 and hapten 2 were used as immunizing haptens. To attempt to produce specific antibodies against both types of pyrethroid insecticides, a mixture of conjugates of hapten 1 and hapten 2 was also regarded as an immunogen. All of the above OVA conjugates, (OVA-hapten 1 (CAg-1), OVA-hapten 2 (CAg-2), OVA-hapten 4 (CAg-4), OVA-hapten 5 (CAg-5), OVA-hapten XQ (CAg-XQ)) were used as coating antigen. According to references, some generic immunizing haptens of each type of pyrethroids have been described [7,31,32,33]. Comparing with the reported, both hapten 1 and hapten 2 retain the ester group but lose a cyclopropane group. 

### Effect of the haptens on affinity of the antisera for the coating antigens

The most important precondition is that a suffcient titer value for combination of antibody and antigen exist [34]. To investigate homo- and heterologous affinity, twenty antibody/coating conjugate combinations were tested by a noncompetitive ELISA protocol. Some titer values, which were found to be difference among the combinations are shown in Table 1.

**Table 1:** The titer values of antisera (absorbance at 450 nm).^ a^

Immunogen	Antiserum	Antibody dilution	Coating antigen (1 μg mL^-1^)				
			cAg-1	cAg-2	cAg-4	cAg-5	cAg-XQ
BSA-hapten 1	pAb-1 ^b^	1: 8000	1.65 1	0.398	0.629	0.412	0.257
BSA-hapten 2	pAb-2 ^b^	1: 8000	0.573	1.484	0.384	0.198	0.626
BSA-hapten 1	pAb-m1 ^c^	1: 3200	1.344	0.925	0.754	0.458	0.174
+ BSA-hapten 2	pAb-m2 ^c^	1: 3200	1.302	1.064	0.400	0.352	0.174

^a ^Four antibodies, named pAb-1, pAb-2, pAb-m1, and pAb-m2 respectively, were determined. Absorbances were measured by a checkerboard pattern with several coating conjugate concentrations and several antibody dilutions, and measured after a 12-min incubation with TMB at 37 °C. For convenience, only data from a coating antigen concentration of 0.1 μg per well and an antibody dilution of 1:8,000 or 1:3,200 are shown. Titer values are the means of three replicates.

The results indicated that the antibodies induced by type I hapten (hapten 1) had cross-reactivity to type II hapten (hapten 2) and the reverse was also true. Considering the distguishing titer difference between coating haptens with and without a cyano group, we thought that the cyano group had a strong effect on such a antibody/hapten reaction system. We also discovered significantly different data for the combination of pAb-2/cAg-1 (0.573) and of pAb-2/cAg-4 (0.384), which indicated that the ester group had also little effects on the reaction system. 

The results of those antibodies against the mixture immunogen (BSA-hapten 1 and BSA-hapten 2) showed that the combination of pAb-m1/cAg-4 had a high titer value (0.745) in addition to “semi-homologous combinations”, such as pAb-m1/cAg-1, pAb-m1/cAg-2 and so on. Those combinations of antibody/coating antigen, with enough affinity and possibility to develop an ELISA for both types of synthetic pyrethroid insecticides, were selected for further studies.

### Effect of the haptens on ELISA sensitivity and specificity

To investigate homo- and heterologous sensitivity, five antibody/coating conjugate combinations were tested by a noncompetitive ELISA protocol. To screen the broadest specific combination, five synthetic pyrethroid insecticides were select for cross-reactivity analysis. The sensitivity and specificity data are shown in Table 2.

In the first combination of pAb-1/cAg-2, the ELISA was only sensitive to type I pyrethroids (phenomethrin and permethrin). Nevertheless the ELISA showed more sensitivity to type II (datamethrin, cypermethrin, and cyhalothrin) than to type I in the second combination of pAb-2/cAg-1. Considering that pAb-1 was prepared with type I immunogen and that pAb-2 was prepared with type II, we could obviously see the cyano group on the type II immunogen had an important effect during the immunization process. The result suggested that, with only one type pyrethroid immunogen, whether type I or type II, it is difficult (even impossible) to obtain antibodies with equal sensitivity to both types of pyrethroids.

Therefore, to obtain the antibodies with equal sensitivity to both types of pyrethroids, a mixed immunogen with type I and type II pyrethroid conjugates was used for antibody preparation. In Table 2, the last three combinations were all against such mixed immunogen. The ELISA of pAb-m1/cAg-1 combination showed an I_50_ range of 0.1–0.3 µg mL^-1^, pAb-m1/cAg-2 combination 0.2–0.6 µg mL^-1^, and pAb-m2/cAg-4 combination 0.016–0.023 µg mL^-1^. According to these results we found the last combination of pAb-m2/cAg-4, with a heterologous ELISA system, had the most equal sensitivity (all I_50_s were around 0.02 µg mL^-1^) to both types of pyrethroids, and the sensitivity was close to the references (0.02–0.03 µg mL^-1^ for type I, 0.0015–0.013 µg mL^-1^ for type II) [7,31,32]. So the combination of pAb-m2/cAg-4 was select for further research.

**Table 2:** Specificity of antibody/coating antigen combinations.

Antibody/coating antigen	Pesticide	I_50_ µg mL^-1^	Cross-reactivity % ^b^
pAb-1/cAg-2	phenomethrin	0.29	100
	permethrin	0.42	69.0
	datamethrin	- ^c^	<0.1
	cypermethrin	- ^c^	<0.1
	cyhalothrin	- ^c^	<0.1
pAb-2/cAg-1	phenomethrin	0.204	100
	permethrin	0.325	62.8
	datamethrin	0.052	392.3
	cypermethrin	0.049	416.3
	cyhalothrin	0.058	351.7
pAb-m1/cAg-1	phenomethrin	0.278	100
	permethrin	0.333	83.5
	datamethrin	0.136	204.4
	cypermethrin	0.098	283.7
	cyhalothrin	0.165	168.5
pAb-m1/cAg-2	phenomethrin	0.247	100
	permethrin	0.324	76.2
	datamethrin	0.495	49.9
	cypermethrin	0.631	39.1
	cyhalothrin	0.386	64.0
pAb-m2/cAg-4	phenomethrin	0.017	100
	permethrin (Trans/Cis)	0.022	77.3
	permethrin (Trans)	0.048	35.4
	datamethrin	0.023	73.9
	cypermethrin	0.019	89.5
	cyhalothrin	0.016	106.2
	esfenverate (1S,2S)	0.186	9.1
	fenverate (1R/S,2R/S)	0.125	13.6
	Tetramethrin	>10	<0.1
	Bifenthrin	>10	<0.1
	3-phenoxybenzoic acid	>1	<1

^a ^The coefficient of variation(CV) was below 12%; ^b^ The cross-reactivity of phenomethrin in all combinations was regarded as 100%, and those of other pyrethroids were calculated as follows: cross-reactivity (%) = [I_50_ (phenomethrin) /I_50_ (other pyrethoid)] × 100; ^c^ The data was not calculated because no significant inhibition was observed.

### Average competitive curve and spiked sample analysis

**Figure 2:**
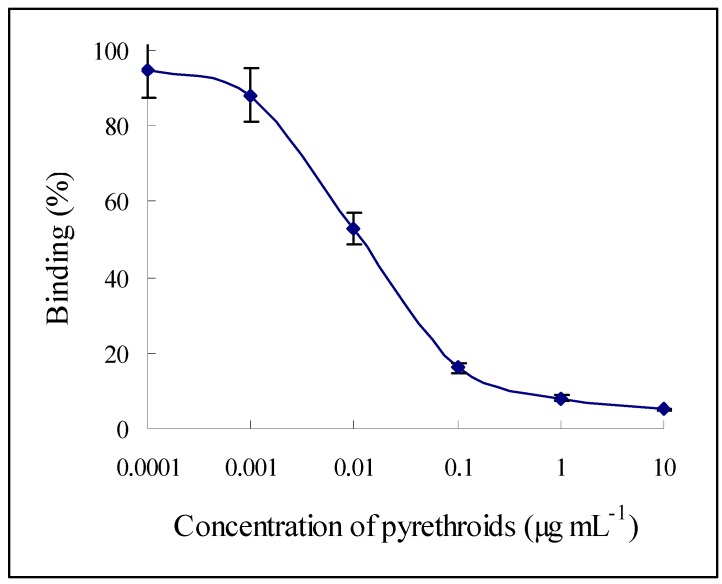
Average standard inhibition curve of five pyrethroids. In an ELISA system, the coating antigen cAg-4 was used with a concentration of 2 µg mL^-1^, and the polyclonal antibody pAb-m1 was used with a dilution of 1:4,000. Then a series concentrations of each standard pyrethroid phenomethrin, permethrin, datamethrin, cypermethrin, and cyhalothrin was tested, whose results contributed to the average standard curve (the average maximum absorbance was 0.952, and the slope value is -1.266). And the average coefficient of variation (CV) was 16%, I_50_ was 0.02 µg mL^-1^, and the dynamic range (I_20_-I_80_) was 0.002–0.084 µg mL^-1^.

In the above selected ELISA system, the coating antigen cAg-4 and the polyclonal antibody pAb-m1 were used. A series of concentrations of each standard pyrethroid: phenomethrin, permethrin, datamethrin, cypermethrin, and cyhalothrin was tested, whose results contributed to the average standard curve (Figure 2). Among the results, the average coefficient of variation (CV) was 16%, I_50_ was 0.02 µg mL^-1^, and the dynamic range (I_20_-I_80_) was 0.002–0.084 µg mL^-1^.

With the average standard curve, a spiking experiment was carried out for an elementary accuracy evaluation. The results showed the recoveries obtained by standard pyrethroids added to tap water samples were from 57% to 73% (Table 3), which indicated that the combination of pAb-m2/cAg-4 will be a useful screening test system for both type I and type II pyrethroids.

Theoretically, we also paid attention here to whether the total concentration of several different compounds with a same type could be calculated as that of just one analyte in a class specific ELISA curve. However, the results could not give clearly an answer to this, so we need to do more related work to answer this question.

**Table 3:** Recovery test of synthetic pyrethroids in drinking water.

pyrethroid insecticide	spiked (µg mL^-1^)	theoretical (µg mL^-1^)	found (µg mL^-1^)	average recovery ± SD (%)
	0	0	< 0.002	-
permethrin	0.01	0.006	0.004	73.3 ± 14.2
datamethin	0.02			
permethrin	0.01	0.010	0.006	56.9 ± 10.6
datamethrin	0.02			
cyhalothrin	0.02			
permethrin	0.02	0.040	0.029	72.9 ± 6.9
datamethin	0.04			
cyhalothrin	0.04			
phenomethrin	0.10			
permethrin	0.02	0.080	0.053	66.3 ± 13.2
datamethin	0.04			
cyhalothrin	0.04			
phenomethrin	0.10			
cypermethrin	0.20			

## Experimental

### Reagents and materials

Chemical reagents for hapten synthesis and pesticide standards used for cross-reactivity studies were supplied by China Redsun Group Nanjing No.1 Pesticide Co., Ltd. (Nanjing, China) and Jiangsu Pesticide Research Institute (Nanjing, China). Analytical-grade solvents were from Sinopharm Group Chemical Reagent Co., Ltd. (Shanghai, China). Tween 20, *N*-hydroxysuccinimide (NHS), *N*,*N*-dicyclohexylcarbodiimide (DCC), tetramethylbenzidine (TMB), peroxidase-labeled goat anti-rabbit immunoglobulins (GAM-HRP), bovine serum albumin (BSA), ovalbumin (OVA), and complete and incomplete Freund’s adjuvants were purchased from Sigma-Aldrich (St. Louis, MO, USA). All other reagents were analytical grade. Glass sheets precoated 0.3-mm silica gel 60 F_254_ for thin-layer chromatography (TLC) and silica gel (60–230 mesh) for column chromatographic purifications were purchased from Qingdao Haiyang Chemical Co., Ltd (Qingdao, China).

### Instruments

Mass spectra were obtained on a HPLCMS-LTQ XL spectrometer (Thermo-Fisher, USA). ^1^H- nuclear magnetic resonance (NMR) spectra were obtained with an INOVA-600 MHz spectrometer (Varian, Palo Alto, CA, USA). Chemical-shift values were given in parts per million (ppm) downfield from the internal standard deuterium chloroform. Coupling constants are expressed in Hz and the abbreviations s, d, t, m, and Ar represent singlet, doublet, triplet, multiplet, and aromatic, respectively. UV-Vis spectra were recorded on a Beckman 640 spectrophotometer. Polystyrene 96-well microtiter plates were from Costar (Corning, MA, USA). A Wellwash 4MK-2 microplate strip washer (Thermo Electron Corporation.) was used to wash ELISA plates. Absorbance (*A*) was measured using a microplate reader (Wallac 1420 Victor 3, Perkin Elmer Inc.).

### Hapten synthesis and verification

Five haptens were mentioned in this paper. The synthetic routes for haptens 1, 2, 4, and 5 are illustrated in Scheme 1. The structure of our previously prepared hapten XQ [35] is also shown in Scheme 1.

*4-Oxo-4-(3-phenoxybenzyloxy)butanoic acid (hapten 1):* Succinic anhydride (0.7 g, 6 mmol) was added to a solution of (3-phenoxyphenyl)methanol (1.2 g, 6 mmol) in dichloromethane (100 mL). After stirring at room temperature overnight, the solution was washed with water and then dried over anhydrous sodium sulfate. Finally, the solution was concentrated and gave hapten 1 (1.6 g, 94%) as a white solid. ^1^H-NMR (CD_3_Cl) δ: 2.66-2.68 (t, 2H, CH_2_), 2.69-2.70 (t, 2H, CH_2_), 6.94-7.36 (m, 9H, Ar); MS (ESI) *m/z* (%): 285 (M-H^+^, 89).

*4-(Cyano(3-phenoxyphenyl)methoxy)-4-oxobutanoic acid (hapten 2):* The aldehyde 3-phenoxybenzaldehyde (1.2 g, 6 mmol), in THF (9 mL) and water (1 mL), was cooled in ice. Powdered potassuim cyanide (0.2 g, 6 mmol) was added into the solution of the aldehyde. With stirring, 24 N HCl (0.4 mL) was dropped slowly into the mixture. The reaction went on for 40 min, and then the mixture was acidified with 3 N HCl and extracted with ether, and the organic phase was washed with water, dried over anhydrous magnesium sulfate, and evaporated to give the cyanohydrin, 2-hydroxy-2-(3-phenoxyphenyl)acetonitrile, as a brown oil. Subsequently the brown oil was used to synthesize hapten 2 by the same method as used for hapten 1. Finally, 1.6 g of hapten 2 was obtained as a white solid, giving a yield of 87%. ^1^H-NMR (CDCl_3_) δ: 2.68-2.70 (t, 2H, CH_2_), 2.72-2.75 (t, 2H, CH_2_), 5.30 (s, 1H, Ar-CH), 7.03-7.41 (m, 9H, Ar); MS (ESI) *m/z* (%): 310 (M-H^+^, 62).

*3-(3-Phenoxybenzyloxy)propanoic acid (hapten 4):* To a solution of (3-phenoxyphenyl)methanol (1.0 g, 5 mmol) in dry acetone (30 mL) were added anhydrous potassium carbonate (0.6 g, 5 mmol) and ethyl bromoacetate (0.8 g, 5 mmol). After refluxing for 15 h, the mixture was filtered and the solvent was evaporated under reduced pressure. The residue was dissolved in ethyl acetate, washed with cold water, 1 M NaOH and 4 M NaCl, and dried over anhydrous sodium sulfate. Removal of the solvent gave ethyl 3-(3-phenoxybenzyloxy)propanoate as a yellow oil. The ester was dissolved in THF (2 mL) and 1 M NaOH (12 mL) was added. After refluxing for 2 h, the mixture was extracted with CH_2_Cl_2_. The aqueous layer was acidified to pH 3-4 by careful addition of concentrated HCl and then extracted with ethyl acetate. The organic layer was dried over anhydrous sodium sulfate and concentrated to give hapten 4 (0.7 g, 52%) as a white solid. ^1^H-NMR (acetone-*d*_6_) δ: 2.01-2.02 (t, 2H, CH_2_-O), 2.16-2.17 (t, 2H, CH_2_-C), 3.38 (s, 2H, Ar-CH_2_), 6.99-7.39 (m, 9H, Ar); MS (ESI) *m/z* (%): 285 (M-H^+^, 66).

*3-(3-Phenoxybenzamido)propanoic acid (hapten 5):* In a solution of anhydrous DMF (3 mL), 3-phenoxybenzoic acid (1.1 g, 5 mmol), NHS (0.6 g, 5 mmol) and DCC (1.0 g, 5 mmol) were added. With stirring, the reaction went on for 3 h at room temperature. The mixture was stored at 4 °C overnight, then the supernatant containing the active ester, 2,5-dioxopyrrolidin-1-yl 3-phenoxybenzoate, was separated and slowly added dropwise to a solution of 3-aminopropanoic acid (0.5 g, 5 mmol) in PBS (12 mL). The reaction was carried on for 1 h at room temperature and then overnight at 4 °C. After removal of the solvent, the residue was dissolved in ethyl acetate, washed with cold water, 1 M NaOH and 4 M NaCl, and dried over anhydrous sodium sulfate. Final removal of the solvent gave hapten 5 (0.8 g, 57%) as a white solid. ^1^H-NMR (acetone-*d*_6_)δ: 2.24-2.27 (m, 1H, CH_2_), 2.02-2.06 (t, 2H, CH2-C), 7.05-7.79 (m, 9H, Ar), 10.83 (s, 1H, NH); MS (ESI) *m/z* (%): 284 (M-H^+^, 32).

### Preparation of immunogens and coating antigens

The conjugations of the five haptens above and the estimations of hapten densities were carried out according to Zhang [36]. To generate immunogens, haptens 1 and B were covalently attached through their carboxylic acid moieties to the lysine groups of BSA using the active ester method. Using the same method, haptens 1, 2, 4, 5, and XQ were coupled to OVA to obtain coating antigens. The immunogens and coating antigens were purified by dialysis in phosphate buffer (PB: 0.02 mol L^-1^, pH 6.8). The conjugates were stored at -20 °C until use. UV-Vis spectral data were used to confirm the structures of the final conjugates. Assuming that the molar absorptivity of haptens was the same for the free and conjugated forms, the hapten densities (the number of hapten molecules per molecule of protein) of the conjugates were estimated directly by the mole absorbance *ε*:

Hapten density = (*ε*_conjugation_ -*ε*_protein_ )/*ε*_hapten_

### Immunization

Four female New Zealand white rabbits of about three months of age were immunized with the conjugates of BSA-hapten 1, BSA-hapten 2, or the mixture of equal amount of BSA-hapten 1 and BSA-hapten 2. The first dose consisted of 800 μg of conjugate injected as an emulsion of PBS and Freund’s complete adjuvant. Three subsequent injections emulsified in Freund’s incomplete adjuvant were given at three-week intervals. One week after the last injection, the rabbits were bled, and the production of Ab was made following the protocol reported by Shan [23]. The anti-hapten antibody titers of the sera were tested by indirect ELISA, and the analyte recognition properties were examined by competitive indirect ELISA. Four sera were obtained and tested: pAb-1 against BSA-hapten 1, pAb-2 against BSA-hapten 2, and pAb-m1 and pAb-m2 against the mixture immunogen.

### Titration of antisera

The titers of antisera were determined by measuring the binding of serial dilutions of the antisera to the corresponding coating antigen (hapten-OVA) using noncompetitive ELISA protocol. Polystyrene microtiter plates were coated with the coating antigen (1 μg mL^-1^, 50 μL per well) in 50 mmol L^-1^ carbonate-bicarbonate buffer (pH 9.6) by 2 h incubation at 37 °C. The following steps were the same as the description of Zhang [33]. The coated plates were washed five times with PBST (PBS containing 0.05% Tween 20, pH 7.4) and blocked by incubation with 1% gelatin in PBS (100 μL per well) for 1.5 h at 37 °C. After another washing step, 50 μL per well of antiserum diluted with PBS (1:1,600–1:512,000) were added to the plate, and the plates were incubated for 1 h at 37 °C. After another washing step, 50 μL of a GAM-HRP conjugate diluted 1:10,000 with PBST were added to each well and incubated for 1 h at 37 °C. Next, the plates were washed again, and 50 μL of substrate solution (3.3 μL of 30% H_2_O_2_ and 400 μL of 0.6% TMB in DMSO per 25 mL of acetate buffer, pH 5.5) were added to each well. Color development was stopped after 15-30 min with 25 μL per well of 2 mol L^-1^ H_2_SO_4_. The absorbance was measured using the single-wavelength mode at 450 nm.

### Determination of the effect of the haptens on the affinity of antisera for coating antigens

The affinity of each of the six antisera for each of the five coating antigens (hapten-OVA conjugates) was determined by noncompetitive indirect ELISA as follows: all incubations were performed at 37 °C, including the incubation of 2 h with the coating antigens. Microtiter plates were coated with the hapten-OVA conjugates (25, 50, 100, or 200 ng per well), and 50 μL of antiserum was diluted with PBST (1:1,600, 1:3,200, 1: 6,400, 1:8,000, 1:12,800, or 1:16,000). The other steps were described as the above.

### Determination of the effect of the haptens on the ELISA sensitivity and specificity

The effect of hapten heterology between the immunogen and the coating antigen on the ELISA sensitivity and specificity was investigated by competitive indirect ELISA for all possible combinations of antiserum and coating antigen. The assay procedure was presented previously [34]. All incubations were performed at 37 °C except for the incubation with the coating antigens. Microtiter plates were coated with hapten-OVA conjugate (1 μg mL^-1^, 50 μL per well) in 50 mM carbonate-bicarbonate buffer (pH 9.6) by overnight incubation at 4 °C. The plates were washed five times with PBST and blocked by incubation with 100 μL per well of 1% gelatin in PBS for 1.5 h. After another washing step, 25 μL per well of serial dilutions of the analyte in 40% methanol-PBS were added, followed by 25 μL per well of antiserum diluted in PBST. After incubation for 1 h, the antibody binding was assessed as described above using HRP-conjugated goat anti-mouse IgG diluted in PBST. I_50_ values (the concentration at which the binding of the antibody to the coating antigen is inhibited by 50%) were determined using logistic equations. The specificities of the ELISAs against several pyrethroid pesticides were determined and calculated as follows: CR (%) = [I_50_ (fenthion)/I_50_ (test compound)] × 100.

### Average competitive curve and water sample analysis

In an ELISA system, the coating antigen cAg-4 was used with a concentration of 2 µg mL^-1^, and the polyclonal antibody pAb-m1 was used with a dilution of 1:4000. Then a series concentrations (0.0001, 0.001, 0.01, 0.1, 1, and 10 µg mL^-1^) of each standard pyrethroid phenomethrin, permethrin, datamethrin, cypermethrin, and cyhalothrin was tested, whose results contributed to the average standard curve.

To simply evaluate the above ELISA, several water samples spiked with different pyrethoid insecticides were prepared. The tap water was collected from local families in Wuhan, Hubei Province. For the spike-and-recovery test, five final concentrations (0, 0.03, 0.05, 0.2, 0.4 µg mL^-1^) of pyrethroids of the above samples were prepared. These Water samples were detected directly by the developed ELISA (the samples were diluted five times with PBS-methanol buffer).

## Conclusions

Usually, a generic hapten was used for preparation of a class specific antibody against a type of anlytes [37]. In this paper, with a mixture of conjugates of type I and type II pyrethroids, we prepared a polyclonal antibody against both types of pyrethroid insecticides. Therefore, use of a mixture immunogen, seems to provide a new behavior for preparation of class specific antibodies against a group of small molecular analytes. In addition, we found the best combination of pAb-m2/cAg-4 with equal high sensitivities (about 0.02 µg mL^-1^) to both types of pyrethroid insecticides tested here, such as phenomethrin, permethrin, datamethrin, cypermethrin, and cyhalothrin. With the developed heterologous ELISA system, the recovery tests show that this assay can be used for screening water samples for pyrethroid multi-residues.
